# *Penicillium simplicissimum* NL-Z1 Induced an Imposed Effect to Promote the Leguminous Plant Growth

**DOI:** 10.3389/fmicb.2021.738734

**Published:** 2021-09-28

**Authors:** Jiayao Zhuang, Chao Liu, Xiaoxue Wang, Tongxin Xu, Hao Yang

**Affiliations:** Collaborative Innovation Center of Sustainable Forestry in Southern China of Jiangsu Province, Nanjing Forestry University, Nanjing, China

**Keywords:** beneficial microbe, growth-promoting, imposed effect, leguminous plant, weathering

## Abstract

It is found effective for phytoremediation of the guest soil spraying method by adding microbes to promote the growth of arbor leguminous plant on a high and steep rock slope. However, its underlying mechanisms remain elusive. Here, some experiments were conducted to explore the multifunctions of *Penicillium simplicissimum* NL-Z1 on rock weathering, nodule growth, and beneficial microbial regulation. The results show that *P. simplicissimum* NL-Z1 significantly increased the release of phosphorus, potassium, calcium, and magnesium from the rock by 226, 29, 24, and 95%, respectively, compared with that of the control. A significant increase of 153% in *Indigofera pseudotinctoria Matsum* nodule biomass, accompanied by an increase of 37% in the leguminous plant biomass was observed in the *P. simplicissimum* NL-Z1 treatment than in the control treatment. Interestingly, even though *P. simplicissimum* NL-Z1 itself became a minor microbial community in the soil, it induced a significant increase in *Mortierella*, which, as a beneficial microbe, can promote phosphate-solubilizing and plant growth. The results suggest that *P. simplicissimum* NL-Z1 could induce an imposed effect to promote leguminous plant growth, which may be conducive to the development of the phytoremediation technique for high and steep rock slope. The study provides a novel thought of using the indirect effect of microbes, i.e., promoting other beneficial microbes, to improve soil environment.

## Introduction

Human activities such as mining and road construction have destroyed a large number of mountains, where high and steep rock slopes can be seen everywhere (Yang et al., 2012; Li et al., 2014). It is a great threat to the natural landscape and the ecological environment. Phytoremediation by the guest soil spraying method is considered as the most promising technology for its cost effectiveness and environmental friendliness, which has been widely used in the world. However, even with the addition of other soil substrates, the lack of water and fertility on the high and steep rock slopes still results in a great reduction of plants, which is often frustrating. Phytoremediation is a challenging task because of the poor site conditions. To overcome this problem, microbial-assisted phytoremediation is being explored, of which screening suitable microbes and exploring their growth-promoting mechanisms are the primary tasks.

Microbes inhabit the soil environment, which can release mineral elements and improve soil environment (Mapelli et al., 2012; Zhu et al., 2014; Tian et al., 2021). Jongmans et al. (1997) found that symbiotic mycorrhizal hyphae translocate dissolved minerals from the isolated micropores directly to their host plants, and the significant degree of fungus’s effect on rock decomposition can even be described as “rock eating.” Their creative work is a milestone in the mineral weathering by microbes. From then on, there have been many reports on the weathering effect of microbes on minerals. Zolfaghari et al. (2021) isolated phosphate-solubilizing bacteria that could help in coping with the drought conditions by increasing root and improving *oak* seedling growth. Wu et al. (2017) found a bacterium, which can promote rock weathering and increase nutrient elements in the environment, therefore, improving soil formation and plant growth. Zeng et al. (2015) explored the expression of related genes of a microbe and revealed the mechanism of its function on solubilizing mineral phosphate in soil and promoting growth of plants.

Leguminous plant is one of the most commonly used tree species in ecological restoration, of which the research and application of rhizobia has already a history of more than 100 years. In recent years, it is still a hot research topic. For example, *Rhizobium radiobacter* LB2 was screened to promote the growth and yield of leguminous crops under field conditions (Verma et al., 2020). Biochar and *Rhizobium* bacteria were used to improve the growth quality of *Robinia pseudoacacia* seedlings (Sun et al., 2020). These studies improved our understanding of the benefits of rhizobia to promote plant growth, but there is little research on the growth promotion of non-rhizobia on leguminous plants.

In addition, changes in the structure of microbial communities are important biological indicators for evaluating soil ecosystems and is currently a hot research topic, and the development of these communities is crucial to the stability of plants (Martine et al., 2009). A study has shown that the endophyte diversity of the leaves can be controlled to have an impact on the survival and health of plants (Chen, 2020). Growing evidence also suggests that plant fitness can be determined by the surrounding microbiome composition (Panke-Buisse et al., 2014; Wubs et al., 2016; Samaddar et al., 2021; Stefan et al., 2021). Therefore, understanding the changes in the structure of microbial communities will help us to deepen the exploration of microbial mechanisms.

**FIGURE 1:**
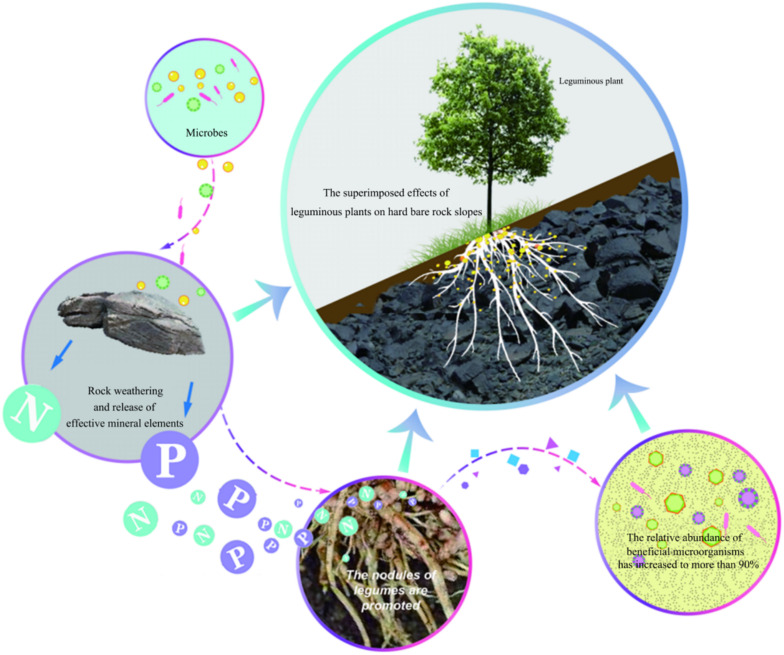
The hypothesis of “the imposed effect for promoting leguminous plant growth.” Some microbes which have the function of weathering rock for releasing mineral elements, promoting the growth of nodule. And based on above, improving the relative abundance of beneficial microbe form 12% to a high level, and finally gives the leguminous plant a cooperative imposed effect to grow well on the high and steep rock slopes by guest soil spraying method.

In previous research, we successfully planted *R. pseudoacacia* on the high and steep rock slopes along the Yueyang Avenue in Hunan, China, by the guest soil spraying method with the addition of microbes (Supplementary Figure 1). Although the arbor trees grow well with the help of microbes on the high and steep rock slopes and form a natural forest landscape, its mechanism is not clear, which hinders further research. So, in our study, we put forward a hypothesis of “the imposed effect for promoting leguminous plant growth” including mineral weathering, nodule growth promotion, and beneficial microbial regulation (Figure 1). To test this hypothesis, we screened strains from the soil sample, and designed weathering experiment and pot experiment to explore the growth-promoting mechanisms of microbes. To understand the soil microbes and its possible effect, the fungi communities in potted soil were investigated through Illumina sequencing technology. This work proved the feasibility and efficiency of microbial-assisted phytoremediation. It provides strain and theoretical guidance for improving the phytoremediation efficiency of high and steep rock slopes and similar barren areas.

## Materials and Methods

### Isolation and Screening

#### Media

*Nutrient agar* (NA, for cultivating bacteria): 3 g of beef extract, 10 g of peptone, 5 g of NaCl, 20 g of agar, and 1,000 ml of deionized water, pH 7.0–7.2.

*Potato dextrose agar* (PDA, for cultivating fungi): 6 g of potato flour, 20 g of glucose, 18 g of agar, and 1,000 ml of deionized water, pH 7.0–7.2.

*Monkina organic phosphorus agar* (for screening strains with the phosphorus-solubilizing effect): 10 g of glucose, 0.5 g of (NH_4_)_2_SO_4_, 0.3 g of NaCl, 0.3 g of KCl, 0.3 g of MgSO_4_⋅7H_2_O, 0.03 g of FeSO_4_⋅7H_2_O, 0.03 g of MnSO_4_, 5.0 g of CaCO_3_, 0.3 g of lecithin, 20 g of agar, and 1,000 ml of deionized water, pH 7.0–7.5.

*Monkina inorganic phosphorus agar* (for screening strains with the phosphorus-solubilizing effect): 10 g of glucose, 0.5 g of (NH_4_)_2_SO_4_, 0.3 g of NaCl, 0.3 g of KCl, 0.3 g of MgSO_4_⋅7H_2_O, 0.03 g of FeSO_4_⋅7H_2_O, 0.03 g of MnSO_4_, 5.0 g of Ca_3_(PO4)_2_, 20 g of agar, and 1,000 ml of deionized water, pH 7.0–7.5.

*LB media* (for the amplification of bacteria): 10 g of peptone, 5 g of yeast, 5 g of NaCl, and 1,000 ml of deionized water, pH 7.2.

*Martin broth* (for the amplification of fungi): 5 g of peptone, 1 g of K_2_HPO_4_, 2 g of yeast, 0.5 g of MgSO_4_, 20 g of glucose, and 1,000 ml of deionized water, pH 6.8.

*Modified Monkina broth media* (for weathering experiment): remove the phosphorus component of Monkina agar, so that the phosphorus only comes from the rock particles added later.

#### Isolation and Screening

The soil samples for screening microbes and rock samples for mineral analysis and weathering experiment were collected from the high and steep rock slopes along the Yueyang Avenue in the Yueyang City of Hunan Province, China.

A 10-fold serial dilution of soil samples was smeared on NA and PDA to isolate bacteria and fungi, respectively. The agar plates were incubated at 28°C for 3 days. Morphologically distinct colonies were subjected to purification following subculturing. The pure cultures were maintained on NA (bacteria) and PDA (fungi) slants at 4°C in a refrigerator. The screening of phosphate-solubilizing microbes was carried out on Monkina agar at 28°C following spot inoculation. The agar plates exhibited the formation of halos (zone of solubilization) around the colonies after 7 days of incubation. Qualitative estimation of the phosphate-solubilizing microbe isolates were conducted through plate assays using spot inoculation on Monkina agar. The diameter of the halo size around the microbe colony (D) and the colony (d) were measured after 7 days of incubation. The value of D/d was calculated by dividing the diameter of the halo size by the diameter of the colony. Based on the result of the value of D/d, five strains of phosphate-solubilizing microbes were selected for weathering experiment.

### Weathering Experiment

Quantitative estimation and exploration of the capability to dissolve rocks through weathering experiment: To make the broth, a 1-mm-diameter disk from activated isolates X-4, X-8, X-11, and X-14 grown on NA and NL-Z1 grown on PDA were added to 100-ml Erlenmeyer flasks containing 30 ml of LB media (for X-4, X-8, X-11, and X-14) or Martin broth (for NL-Z1) and incubated at 30°C and 180 rpm for 3 days, respectively. Then 1 ml of the broth of five strains were inoculated into a 30-ml modified Monkina broth media (contains 1.5 g of rock particles, which replaced the phosphorus component of Monkina agar) in an Erlenmeyer flask and incubated at 30°C and 180 rpm for 10 days. Samples (*n* = 3) from the inoculated groups (X-4, X-8, X-11, X-14, and NL-Z1) and control group were collected at different intervals (4, 7, and 10 days), centrifuged at 8,000 rpm for 10 min. Five milliliters of the supernatant was filtered through a 0.2-μm membrane filter into a centrifuge tube for the determination of pH, available phosphorus (AP), potassium (K), calcium (Ca), and magnesium (Mg). pH was determined using the METTLER TOLEDO pH meter. AP was determined using the molybdenum–antimony anticolorimetric method. K, Ca, and Mg were determined using the atomic absorption spectrometer. The shape and size of the rock particles were measured at the last sampling (10 days).

### Pot Experiment

Based on the results of the weathering experiment, the strain NL-Z1 was selected, and its capability of promoting growth through pot experiment was explored.

For the production of fungus NL-Z1 inocula, the isolate was grown on PDA until the mycelia produced conidia. Spores were added to 100-ml Erlenmeyer flasks containing 30 ml of Martin broth and incubated at 30°C and 180 rpm for a respective number of days. In this process, the absorbance of the suspension was measured (UV-8000T, Shanghai Metash Instruments Co., Ltd.) at 600 nm (Ibiang et al., 2020), and it was made sure that the absorbance was in the range of 0.8∼1.2 by diluting or continuing to incubate the suspension of inocula. The inocula of NL-Z1 was sealed and stored in a refrigerator at 4°C for later use. At the time of inoculation, the stored suspension of inocula was diluted 100 times, and 60 ml of it was applied to the soil.

To explore the growth-promoting effect, the fungal inocula was applied on the seedlings of *I. Matsum*. To do this, the seeds of *I. Matsum* were surface-sterilized and then germinated (3 days, 20°C, and relative humidity of 60%), and then three healthy seedlings were planted in each pot including mixed matrix soil (provided by Jiangsu Xingnong Matrix Technology Co., Ltd). One month later, one seedling was kept per pot (ensure that the growth of seedlings in each pot is basically the same). The pots were divided into two groups (CK and NL-Z1, three replicates for each group) and placed in a greenhouse. Subsequently, the CK group was added with the sterile culture solution, and the NL-Z1 group was added with the same amount of the suspension of inocula.

Three months later, the plants were collected, and the indices were measured. For plants, vernier calipers and tape were used to measure the ground diameter and height of the seedlings, a root scanner was used to measure the leaf area (a total of 10 upper, middle, and lower leaves are selected for each pot to measure the leaf area), and the plants were dried to measure the aboveground and underground biomass. For potted soil, the METTLER TOLEDO pH meter was used to determine its pH (water–soil ratio is 5:1). The molybdenum–antimony anticolorimetric method was used to determine the soil AP. Alkaline hydrolysis (HN) diffusion method was used to determine the soil hydrolyzed nitrogen (HN).

### Strain Identification and Metagenomic Sequencing

The fungus NL-Z1 was identified based on morphological characteristics and ITS rRNA analysis. The molecular level identification was carried out with the support of the Shanghai Jinyu Medical Laboratory Co., Ltd. The sequence obtained was uploaded to the GenBank database^1^. The potted soil samples were sent to Shanghai Majorbio Bio-pharm Technology Co., Ltd., for genetic sequencing. Microbial DNA was extracted using the HiPure Soil DNA Kits according to the protocols of the manufacturer. The ITS gene was amplified by PCR (94°C for 2 min, followed by 30 cycles at 95°C for 1 min, 60°C for 1 min, and 72°C for min and a final extension at 72°C for 7 min) using ITS3_KYO2 primer (5′-GATGAAGAACGYAGYRAA-3′) and combined ITS4 primer (5′-TCCTCCGCTTATTGATATGC-3′) targeting the ITS2 region. PCR reactions were performed in triplicate 50-μl mixture containing 10 μl of 5 × Q5@ Reaction Buffer, 10 μl of 5 × Q5@ High GC Enhancer, 1.5 μl of 2.5 mM dNTPs, 1.5 μl of each primer (10 μM), 0.2 μl of Q5@ High-Fidelity DNA Polymerase, and 50 ng of template DNA. Related PCR reagents were from New England Biolabs, United States. AMPure XP Beads were used to purify the second round of amplified products, The ABI StepOnePlus Real Time PCR System (Life Technologies, produced in the United States) was used for quantification. Sequencing was performed on a computer based on the PE250 mode pooling of NovaSeq 6000.

### Sequencing Data Analysis and Registration

The original sequence data and sequencing quality files were obtained from FASTA files. According to Schloss (2009), the Mothur software was used to access the files for processing and analysis. In order to illustrate the microbial diversity of potted soil and the abundance of dominant species, the α diversity index and relative abundance (including Chao1, Simpson, and Shannon indices) were quantified by OTU abundance. R’s default ggplot2 package was used to make species composition analysis graphs and vegan package for environmental correlation analysis, which can detect the relationship between environmental factors, samples, and flora.

The isolate of NL-Z1 was deposited in the *China Center for Type Culture Collection* with the deposit number M2019999; the nucleotide sequence of NL-Z1 and the potted soil ITS sequence were uploaded to the NCBI database, and the registration numbers are MZ348581 and SRP322824, respectively.

## Results and Analysis

### Isolation and Screening

A total of 58 strains were isolated in this study, of which 21 strains were verified to be able to solute phosphate (Table 1). According to the results of qualitative estimation, five strains of phosphate-solubilizing microbes were selected for weathering experiment. They were named X-4, X-8, X-11, X-14, and NL-Z1.

**TABLE 1:** Primary effect of phosphate-dissolving bacteria, Unit: cm.

**Name**	**D/d (organic)**	**D/d (inorganic)**	**Name**	**D/d (organic)**	**D/d (inorganic)**
X-4	3.61a	2.13c	X-35	–	1.27f
X-8	–	2.60bc	X-38	2.41bc	1.93c
X-11	3.06ab	4.54a	X-42	–	1.57d
X-14	3.47a	2.81b	X-43	2.83b	–
X-17	2.22c	–	X-44	1.42e	–
X-19	2.09cd	2.60bc	X-48	2.25c	–
X-25	2.49b	1.53d	X-53	1.79d	–
X-27	2.50b	2.00c	X-55	2.22c	1.37f
X-30	–	1.69d	X-58	2.17c	–
X-33	–	1.50d	NL-Z1	3.75a	1.55d
X-34	–	1.44df			

*The values “d, D” represent the diameter of the colony and the halo size around the microbe colony, respectively. Different letters (a, b, c, d, e, f) represent significant differences (*p* < 0.05).*

### The Weathering Effect

**FIGURE 2:**
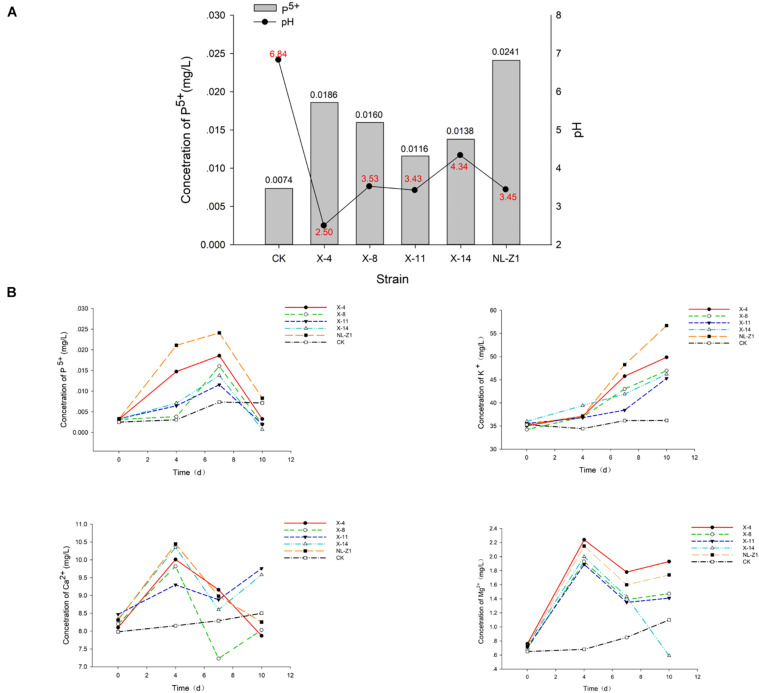
Peak release of available phosphorus concentration **(A)** and concentration dynamic changes of each element in the weathering experiment **(B)**.

**FIGURE 3:**
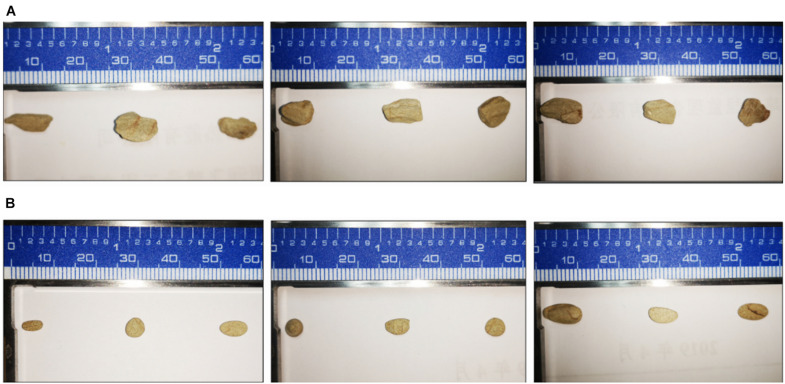
The dissolution of the rock after 10 days by *P. simplicissimum.* **(A,B)** Three replicates in the NL-Z1 treatment group before and after the experiment, respectively.

**FIGURE 4:**
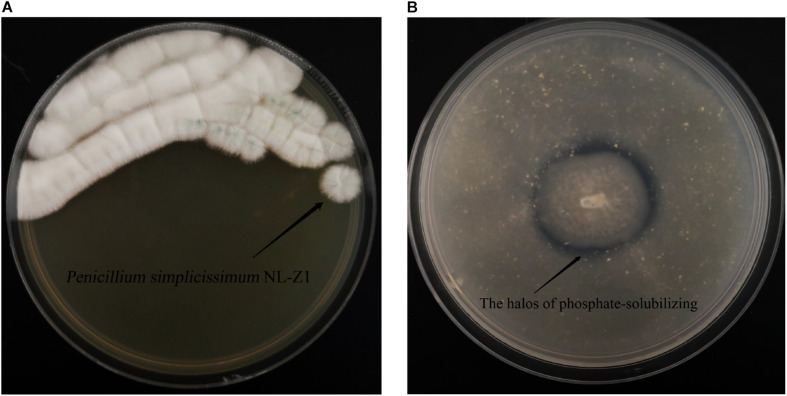
*Penicillium simplicissimum* NL-Z1 **(A)** and its halos on Monkina agar **(B)**.

According to the results of mineral analysis, the main components of rock samples include the following: K_2_O 3.71%, Na_2_O 1.39%, CaO 0.21%, MgO 1.28%, P_2_O_5_ 0.11%, Fe_2_O_3_ 6.81%, Al_2_O_3_ 15.21%, and MnO 0.04%. Visible from Figure 2A, the five strains all promoted the release of AP in the rock to a certain extent. Among them, fungus NL-Z1 has the largest release peak, and its concentration is 0.0241 mg/L, which is about 3.26 times that of the control group, and the pH of each inoculated group decreased. Compared with other inoculated groups, NL-Z1 has a stronger capability to release various elements, and the peak release of K, Ca, and Mg increased by 29, 24, and 95%, respectively (Figure 2B). In general, the release of P, K, Ca, and Mg in rocks by each strain shows an upward trend first, and then a downward trend. After the end of the experiment, the appearance of the rock particles changed dramatically, and the rock was obviously smaller and very smooth (Figure 3). The results of quantitative estimation showed that NL-Z1 maintains a good release effect for each element, the shape of the rock changes significantly, and it has the best rock weathering capability. Since this study only focused on the strain with the best weathering capability, only the fungus NL-Z1 was identified, and it was identified as *P. simplicissimum.* The characteristic morphology of the hypha in the PDA plate is shown in Figure 4A. A clear zone of solubilization of strain in the Monkina agar plate shows the high phosphate-solubilizing ability of NL-Z1 (Figure 4B).

### The Growth-Promoting Effect

The nodule is the primary concern in the research process of leguminous plants. Table 2 shows the plant growth and soil properties. The control group formed an average of eight nodules with a total nodule weight of 0.19 g; the *I. Matsum* seedlings treated with strain NL-Z1 formed an average of six nodules, with a total nodule weight of 0.48 g. Compared with the former, the number of nodules in the latter equine spine plants was slightly lower, but the total mass of the nodules was significantly increased by 153% (*p* < 0.05). In addition, in the control group, the root biomass, root surface area, and root volume of *I. Matsum* were 1.20 g, 232.26 cm^2^, and 1.71 cm^3^, respectively; in the NL-Z1 group, the root biomass was 1.60 g, which was significantly increased by 33% (*p* < 0.05), the root surface area was 246.53 cm^2^, with an increase of 6%, and the root volume was 2.12 cm^3^, with an increase of 24%. Next, the aboveground indicators of the NL-Z1 group are higher than those of the control group. The average aboveground biomass was 7.90 g, with a significant increase of about 38% (*p* < 0.05); the average ground diameter was 5.02 mm, with a significant increase of about 10% (*p* < 0.05); the average seedling height was 74.67 cm, with a significant increase of about 37%. The average leaf area is 6.25 cm^2^, with a significant increase of about 25% (*p* < 0.05). Finally, the AP and HN concentrations of the potted soil were 3.360 and 238.83 mg/kg, respectively, in which the AP concentrations increased significantly by 37% (*p* < 0.05), and the HN increased by 15%. The potted soil has a certain degree of acidification, and the pH is reduced from 7.06 to 6.79.

**TABLE 2:** Effects of fungus NL-Z1 on plants and soil.

**Groups (sample)**	**Plant (underground)**			**Plant (aboveground)**				**Soil (potted, mg/kg)**		
	**Total nodule weight (g)**	**Dry weight (g)**	**Root volume (cm^3^)**	**Dry weight (g)**	**Ground diameter (mm)**	**Plant height (cm)**	**Average leaf area (cm^2^)**	**AP**	**HN**	**pH**
CK	0.19 ± 0.06b	1.20 ± 0.07b	1.71 ± 0.02a	5.74 ± 0.07b	4.55 ± 0.39b	54.67 ± 2.52b	5.02 ± 0.30b	2.46 ± 0.36b	207.17 ± 3.21a	7.06 ± 0.06a
NL-Z1	0.48 ± 0.11a	1.60 ± 0.06a	2.12 ± 0.21a	7.90 ± 0.90a	5.02 ± 0.10a	74.67 ± 5.03a	6.25 ± 0.13a	3.36 ± 0.23a	238.83 ± 4.58a	6.79 ± 0.10a

*Different letters (a, b) represent significant differences (*P* < 0.05).*

The pot experiment showed that the strain NL-Z1 can promote the growth of nodules and, at the same time, convert the AP and HN in the soil into a form that can be directly absorbed and utilized by the plant, thereby promoting the growth of the plant. It further verified the growth-promoting effect of non-rhizobium NL-Z1 on *I. Matsum*.

### Soil Fungal Community Responses to Inoculating NL-Z1

**FIGURE 5:**
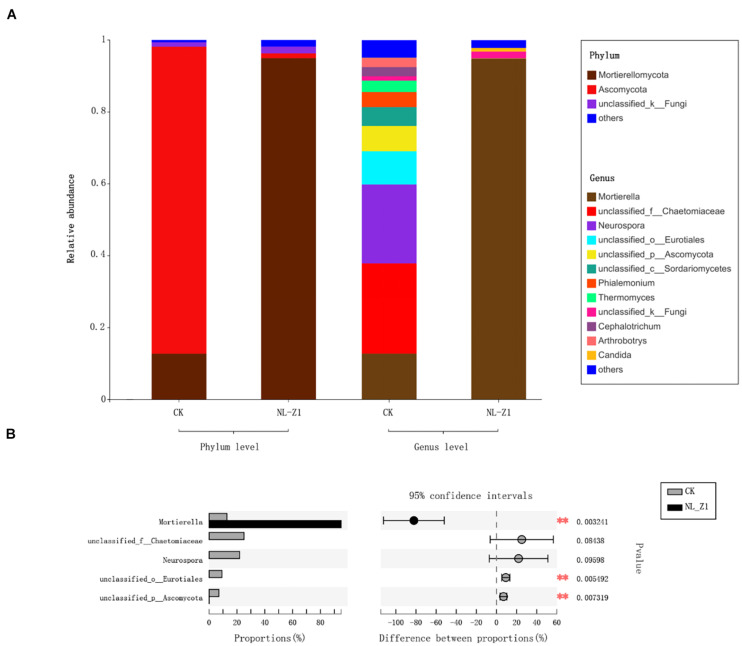
Relative abundance of fungal community at Phylum and Genus level **(A)**. Student’s *t*-test bar plot on Genus level with 95% confidence intervals **(B)**. An asterisk (*) denotes a value significantly differences compared to the corresponding control value according to student’s *t*-test **P < 0.01.

**FIGURE 6:**
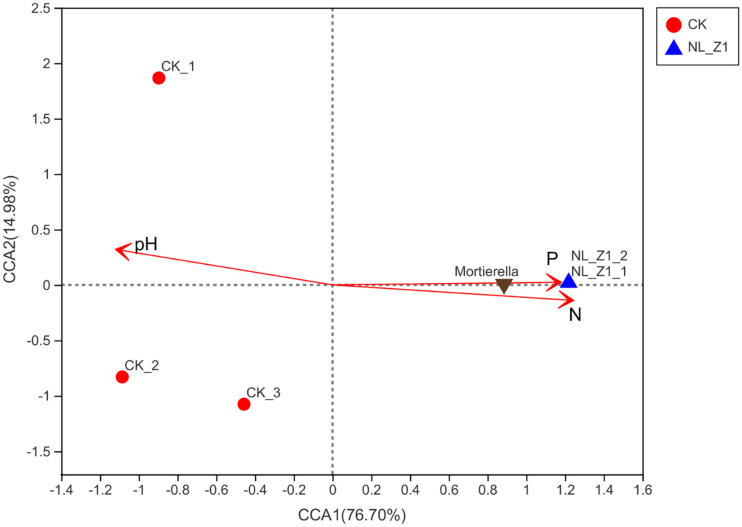
Canonical correlation analysis (CCA) of the fungal communities based on ITS gene amplicon sequencing data. pH (*r*^2^ = –0.8, *P* = 0.23) AP (*r*^2^ = 0.85, *P* = 0.25), and HN (*r*^2^ = 0.95, *P* = 0.16) were significant factors that influenced fungal community.

Figure 5 shows the fungal community structure in potted soil at two levels and Student’s *t*-test bar plot on the genus level. After removing an abnormal sample inoculated with NL-Z1, we found that *Mortierellomycota* (94.95%), instead of *Ascomycota* (85.32%), became the dominant phylum of potted soil fungal communities. *Mortierella* became the dominant genus in the soil community structure, increasing from 12.81 to 94.9% (Figure 5A). We know that *Mortierella* and *P. simplicissimum* NL-Z1 belong to different genera, indicating that the input of NL-Z1 promotes the growth of *Mortierella*. Using the Student’s *t*-test method to test the significance of species differences at the genus level, it was found that *Mortierella* is significantly different between the two groups (*p* < 0.5) (Figure 5B). The results of canonical correspondence analyses (CCA) shows that the samples of the control group and NL-Z1 group were aggregated separately. *Mortierella* has obvious correlation with environmental factors in the soil (AP, *r*^2^ = 0.85, *p* = 0.25; HN, *r*^2^ = 0.95, *p* = 0.16; pH, *r*^2^ = –0.8, *p* = 0.23) (Figure 6). The results suggest that *Mortierella* may be one of the potential reasons for NL-Z1 to improve the soil fertility and exert its growth-promoting benefits.

## Discussion

As the main soil type in southern China, red soil exhibits severe phosphorus deficiency (Kochian, 1995). Therefore, our study is based on phosphate-solubilizing microbes to explore the relevant mechanism. The soil is formed by the long-term weathering process of rocks, in which the cation exchange capacity determines the fertility of the soil. In the weathering experiment, the AP release increased, and the K, Ca, and Mg in the rock were all released, especially in the NL-Z1 group. Tayler et al. (2009) put forward the potential mechanism of the influence of microbes on the process of mineral weathering and pointed out that microbes dissolve minerals through the secretion of H^+^; other studies also show (Balogh-Brunstad et al., 2008) that the effect of acidification of microbes, fungal respiration (CO_2_), and complexation of cation accelerates the weathering of minerals. We found that the pH of the fermentation broth and the potted soil both decreased, indicating that microbes can secrete H^+^ to dissolve minerals. This result is consistent with previous studies. Relevant studies have shown that fungi do not absorb nutrients from minerals through direct contact with hyphae. Acidification is an important driving force for rock weathering. The above analysis reveals that the rock-weathering ability of the fungus NL-Z1 plays an important role in the process of improving the soil and indirectly benefits the growth and development of plants.

As we all know, nodules are formed due to the invasion of rhizobia in the soil into the roots of plants. Rhizobia is related to the development of root nodules and the host itself, but it is not known whether non-rhizobia are also beneficial to the development of leguminous plants. The results of this study show that the inoculation with strain NL-Z1 can significantly promote the growth of *I. Matsum*, and the total weight of nodules increased by 153%. According to reports, nitrogen-containing compounds in the soil will affect the invasion of rhizobia to the host, the development of nodules, and the ability of nitrogen fixation. Too high or too low nitrogen is not conducive to the formation of plant nodules and the effectiveness of plant nitrogen fixation; a moderate nitrogen supply is conducive to the occurrence of nodules (Eaglesham and Szalay, 1983). It can be seen that the increase in *I. Matsum* nodules is inseparable from the improvement of the nitrogen content in the soil by NL-Z1. Another study showed that although there is no clear research conclusion on the effects of phosphorus, nitrogen, and their interaction on plants, to a certain extent, the stimulation of nodulation by phosphorus in the soil can offset the inhibitory effect of nitrogen. Under phosphorus-deficient conditions, the function of nitrogen fixation will be significantly weakened (Luis and Kerstin, 2000). Therefore, the significant increase in the AP content in the soil may be involved in the effective regulation of nitrogen levels, which may also be another important reason for NL-Z1 to promote the growth of *I. Matsum*.

In addition, inoculation with strain NL-Z1 significantly increased the relative abundance of *Mortierella* in the soil. According to previous reports, *Mortierella* can release phosphorus from the soil extensively (Nelson, 2011, 2014). *Mortierella* achieves this function mainly by increasing the levels of soil phosphatase and β-glucase (Li et al., 2018). Therefore, this also implies that the increase in the relative abundance of *Mortierella* may lead to increase in AP in the soil, which is indirectly beneficial to the growth and development of plants. According to the above analysis, NL-Z1, as a kind of phosphate-solubilizing fungi, has the effect of releasing AP, and at the same time, it can synergistically improve the growth of plants by regulating the development of other microbes. Theoretically, it can be considered as the imposed effect of NL-Z1 on the growth promotion of leguminous plants.

Foreign microbial agents will cause changes in the structure of the soil microbial community. In this study, the significant changes in the fungal community were the decrease of *Ascomycota* and the increase of *Mortierellomycota*. Previous studies have shown (Yuan et al., 2020) that the relative abundance of *Ascomycota* is higher in diseased soils, while that of *Mortierellomycota* is higher in healthy soils. Therefore, this is a positive change. In addition, inoculation with NL-Z1 increased the abundance of other genera, especially *Mortierella*, making it the dominant genus in potted soil. Relevant studies have shown (Wubs et al., 2016; Li et al., 2018) that *Mortierella* can effectively improve soil quality and prevent soil degradation. Genetic characteristics show that *Mortierella* has the ability to degrade toxic organic matter, which is beneficial to the health of the soil; *Mortierella* can also significantly increase the level of indole acetic acid in plants and the biomass of plants. A series of studies have strongly proved that *Mortierella* is a beneficial microorganism that is conducive to soil improvement and plant growth. In this study, inoculation of the foreign microbial agent NL-Z1 increased the relative abundance of beneficial microbes in the soil in the short term. Compared with the study of increasing the relative abundance of *Mortierella* through long-term addition of organic fertilizers (Li et al., 2018), it effectively shortened the period of soil improvement. It is a major breakthrough in terms of time.

## Conclusion

Mineral weathering, nodule growth promotion, and microbial regulation are interrelated, which significantly promotes the growth of *I. Matsum* by improving soil quality. In this study, we conducted a 10-day weathering experiment using the screened phosphate-solubilizing strains to explore the ability of soil microbes to decompose rocks and release mineral elements. Our results show that strain NL-Z1 can significantly improve the conversion of insoluble phosphorus and improve soil quality, especially for the improvement of the Red soil region of South China. In addition, a 4-month pot experiment was carried out. Compared with the control, the nodule, dry weight, and the height of the plants inoculated with NL-Z1 increased significantly. By analyzing the properties of potted soil, it is believed that the effect of growth-promoting microbes is inseparable from the improvement of soil fertility. This study also provides a novel thought of using the indirect effect of microbes, i.e., promoting other beneficial microbes, to improve soil quality. Therefore, the hypothesis of the imposed effect of the fungus *P. simplicissimum* NL-Z1 on the growth promotion of leguminous plants is proven. It is of great significance to the improvement of abundance of beneficial microbes in terms of time. The study provides a novel thought of using the indirect effect of microbes, i.e., promoting other beneficial microbes to improve soil environment, which may also provide a revolutionary method for land fertility management.

## Data Availability Statement

The datasets presented in this study can be found in online repositories. The names of the repository/repositories and accession number(s) can be found in the article/Supplementary Material.

## Author Contributions

JZ enabled and supervised the research, conceived and designed the study, and wrote the manuscript. CL, XW, TX, and HY performed the experiments. JZ and CL conducted the data analysis. All authors approved the manuscript.

## Conflict of Interest

The authors declare that the research was conducted in the absence of any commercial or financial relationships that could be construed as a potential conflict of interest.

## Publisher’s Note

All claims expressed in this article are solely those of the authors and do not necessarily represent those of their affiliated organizations, or those of the publisher, the editors and the reviewers. Any product that may be evaluated in this article, or claim that may be made by its manufacturer, is not guaranteed or endorsed by the publisher.

## Footnotes

1
http://www.ncbi.nlm.nih.gov/
