# Supplementary material for: Penicillium simplicissimum NL-Z1 Induced an Imposed Effect to Promote the Leguminous Plant Growth
Source: Front Microbiol. 2021 Sep 28;12:738734. doi: 10.3389/fmicb.2021.738734 (PMC8506219; doi:10.3389/fmicb.2021.738734)
Supplement: Supplementary file 1 [file Data_Sheet_1.docx]

| 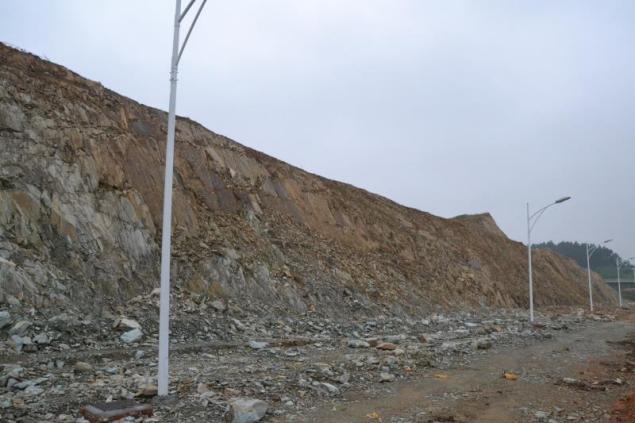 | **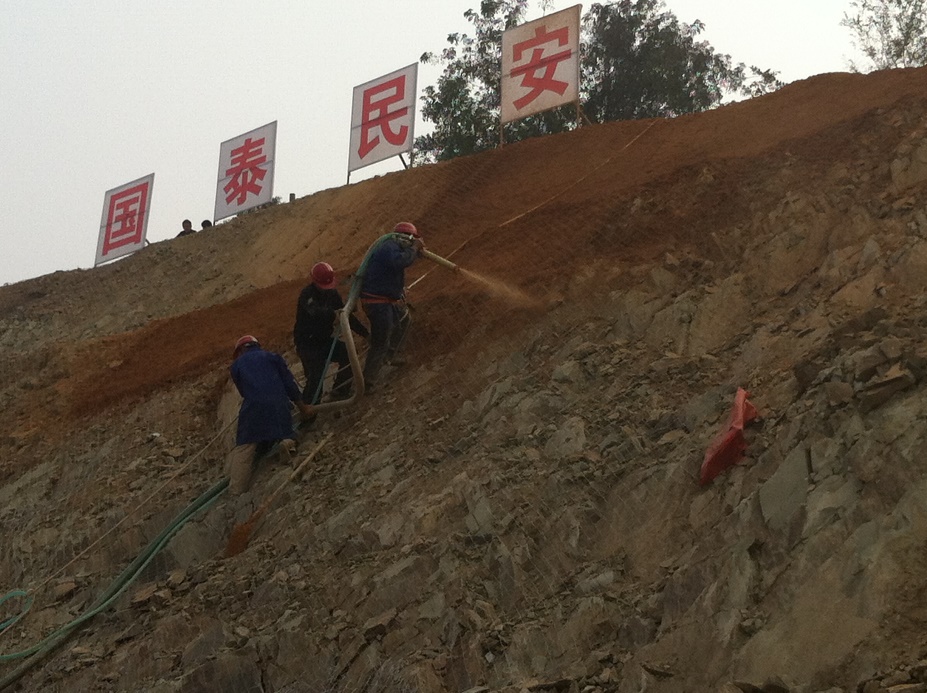** |
| --- | --- |
| **a** | **b** |
| 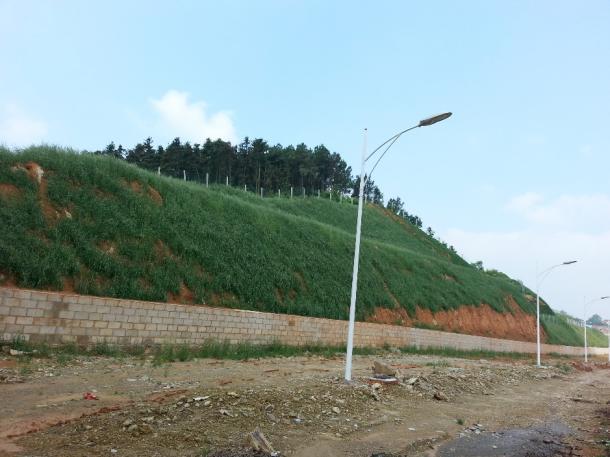 | **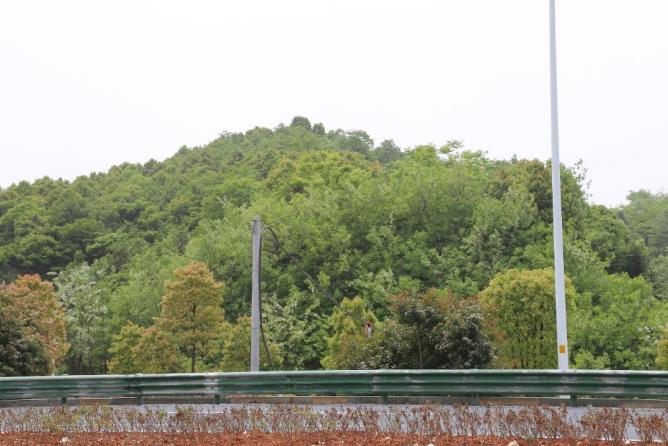** |
| **c** | **d** |
| Fig.S1. Spraying site of high and steep rock slope along the Yueyang Avenue in Hunan, China. (a) refers to the original appearance before spraying. (b) shown the work about spraying. (c) refers to the appearance of the high and steep slope after spraying for two months. (d) refers to the appearance of the high and steep slope after spraying for five years. | |

As can be seen from Fig.S1 that the natural environment of the study site have been restored through the successful application of the guest soil spraying method. And the arbor trees grow well with the help of microbes on the high and steep rock slope and form a natural forest landscape.
